# Synaptic enrichment of microRNAs in adult mouse forebrain is related to structural features of their precursors

**DOI:** 10.1186/1745-6150-3-44

**Published:** 2008-10-29

**Authors:** Neil R Smalheiser

**Affiliations:** 1 Department of Psychiatry and Psychiatric Institute, MC912, University of Illinois at Chicago, 1601 W. Taylor Street, Chicago, IL 60612, USA

## Abstract

Within mouse forebrain, a subset of microRNAs are significantly enriched in synaptoneurosomes (a synaptic fraction containing pinched-off dendritic spines) and a subset are significantly depleted relative to total forebrain homogenate. Here I show that, as a group, the pre-miR hairpin precursors of synaptically enriched microRNAs exhibit significantly different structural features than those that are non-enriched or depleted. Precursors of synaptically enriched microRNAs tend to have a) shorter uninterrupted double-stranded stem segments, and b) more symmetrical bulges containing a single nucleotide on each side. These structural differences may provide a basis for the differential binding of proteins that mediate dendritic transport of pre-miRs, or that prevent pre-miRs from being prematurely processed into mature miRNAs during the transport process.

This article was reviewed by I. King Jordan and Jerzy Jurka.

## Introduction

The brain expresses a wide variety of miRNAs, some of which show regional and cell type specificity [1-6]. miRNAs are also expressed in dendrites where they regulate local protein translation [7,8]. It is uncertain how miRNAs become localized to the dendritic compartment [5,9]. One possibility is that mature miRNAs are formed within the neuronal cell body, and a subset is transported to dendrites in association with their mRNA targets. An alternative hypothesis is that primary miRNA gene transcripts or short hairpin precursors (pre-miRs) might be transported to dendrites in a form that is protected against cleavage.

A recent experimental study of adult mouse forebrain reported the expression of miRNAs in synaptoneurosomes (SYN), a synaptic fraction that is enriched in pinched-off dendritic spines [10]. A significant subset of forebrain-expressed miRNAs (34, or about 14%) is enriched (2-fold or greater) in synaptic fractions relative to total forebrain homogenate, as measured by microarray. These SYN-enriched miRNAs are biologically quite distinct from SYN-depleted miRNAs, both in their expression patterns (many SYN-enriched miRNAs are expressed predominantly in pyramidal neurons, whereas SYN-depleted miRNAs tend to have widespread and abundant tissue expression) and in their evolutionary histories (SYN-enriched miRNAs tend to be evolutionarily new, often mammalian-specific or rodent-specific, whereas the SYN-depleted miRNAs tend to be highly conserved across vertebrates and some had homologues in C. elegans). MiRNA hairpin precursors (pre-miRs) are also detectable in synaptic fractions and postsynaptic densities at levels that are comparable to whole tissue. For seven miRNAs examined, there was a significant correlation between the relative synaptic enrichment of the precursor and the relative synaptic enrichment of the corresponding mature miRNA [10]. Dicer (the RNAse III enzyme that processes pre-miRs to mature miRNAs) and the RISC core Argonaute component eIF2c are also expressed within synaptic fractions and dendritic spines, and dicer is especially enriched in association with postsynaptic densities [11].

These experimental findings suggest that mature miRNAs are formed, at least in part, via processing of pre-miRs locally within dendritic spines [10,11]. As well, the expression of pre-miRs in synaptic fractions implies that the pre-miRs must be transported from the cell body to dendrite shafts and/or to dendritic spines. Yet, currently there is no evidence that pre-miRs are associated with transport complexes within any cell type, nor that the pre-miRs of synaptically enriched miRNAs are preferentially transported to dendrites or to dendritic spines. Can computational analyses provide some insight into this question? If mature microRNAs are the only species that is transported to dendrites, or if pre-miRs are transported in a nondiscriminate fashion, then there would be no reason to expect that the pre-miRs of synaptically enriched vs. non-enriched miRNAs will exhibit any sequence or structural differences. However, if pre-miRs show selective transport, then the pre-miRs of synaptically enriched miRNAs should be demonstrably different from the pre-miRs of non-enriched miRNAs.

As shown in the present report, the set of SYN-enriched miRNAs do exhibit several structural features that distinguish them from miRNAs that show no enrichment, or that are depleted in synaptic fractions relative to the total forebrain homogenate. This provides independent support for the pre-miR selective transport hypothesis, and suggests a basis for differential interaction of pre-miRs with transport complexes.

## Methods

In our previous study, synaptoneurosomes were prepared, characterized and assayed for miRNA expression as described [10]. MiRNAs were measured by microarray to determine the extent of SYN enrichment relative to total forebrain homogenate, together with RT-qPCR validation of selected miRNAs and their precursors. In the analyses described here, the top 20 most SYN-enriched miRNAs were chosen from that study; discarding those having ambiguous or multiple precursor assignments, this gave 17 enriched miRNAs in the "top" set (enrichment ratios = 2.27–4.80 relative to total forebrain homogenate). The next 20 miRNAs were examined similarly, giving 19 enriched miRNAs in the "next" set (enrichment ratios = 1.88–2.24). For comparison, the 20-least enriched miRNAs were examined, again discarding those that can arise from multiple precursors, which gave 15 miRNAs in the "depleted" set (enrichment ratios = 0.15–0.74). Finally, the next 20 least-enriched miRNAs were examined; giving 15 miRNAs in the "not enriched" set (enrichment ratios = 0.76–0.91; these are neither significantly enriched nor depleted relative to total forebrain homogenate). [See Additional File 1 for a list of all miRNAs studied in each set, together with their SYN enrichment ratios.]

**Figure 1:**
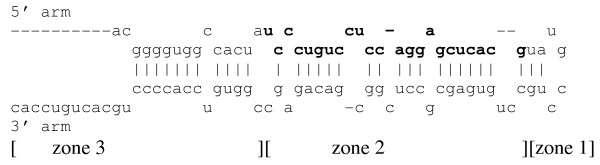
**Diagram showing how a microRNA precursor was divided into zones for scoring.** Shown is pre-miR-339. Zone 1 comprises the loop, zone 2 includes the region giving rise to the mature mir-339 sequence (shown in bold), and zone 3 includes sequences (if any) below the mature microRNA. Regions of bases connected by bonds (including G:U bonds) are referred to as stems, and opposing bases not connected by bonds are referred to as bulges.

Each miRNA (and the predicted folding of its pre-miR) was looked up in miRBase [12] (Release 10.1) in April 2008. Each pre-miR was divided into three zones (containing the loop; giving rise to the mature miRNA sequence; and any additional sequences) and scored for stems and bulges as shown in fig. 1. These were tabulated and scored for features as shown in Additional File 1. The dataset contains several related members of certain families (e.g., let-7b, e, g and i) which were predominantly found in the "not enriched" and "depleted" sets. This might have contributed to a slight systematic bias, but since these arise from different precursors and have nonredundant functions, it was not appropriate to remove such families from the dataset. Note that mir-433-5p was placed in the "top" set whereas mir-433-3p is in the "not enriched" set – thus, the same precursor was scored twice (albeit in relation to the two different mature sequences). A similar situation occurred with mir-30a-3p and 5p that were in the "next" set and the "not enriched" sets, respectively. Conversely, both mir-324-5p and mir-324-3p were placed into the "next" set and both mir-126-5p and -3p were placed into the "depleted" set; in each case, the pairs had very similar enrichment ratios, but only 324-5p and 126-3p were scored, to order to avoid counting the same pre-miR twice within the same sets. Thus, the inclusion of mir-433 and 30a, and the exclusion of 324-3p and 126-5p, is conservative and would possibly UNDER-estimate the true extent of difference between SYN-enriched and non-enriched sets.

## Results

Many of the pre-miR parameters were similar across sets. For example, the average length of zone 1 (the loop) did not differ significantly in the "top" set (13.9 nt) vs. the "depleted" set (14.1 nt), and multiple sequence alignments (of the entire pre-miR sequence or of loop sequences) failed to reveal any recurring motifs characteristic of the "top" set (not shown). When the "top" set was compared to the "depleted" set, a similar proportion of miRNAs arose from the 5' arm of the pre-miR (8/17 vs. 7/15) and a similar proportion of the miRNAs began with a U (11/17 vs. 8/15). Moreover, the first nucleotide of the mature miRNA was found on a bulge (e.g., as in fig. 1) in a similar proportion of miRNAs across sets (8/17 vs. 9/15).

In contrast, significant differences were observed in pre-miRs that related to the stem region (zone 2) encoding the mature miRNA. The total length of stem segments in zone 2 was not significantly different across groups (17.94 nt in "top" set vs. 18.53 nt in "depleted" set). However, the longest uninterrupted stem segment present within the pre-miR was 3 nucleotides shorter on average in the "top" set (8.41 ± 0.68) than in the "depleted" set (11.53 ± 1.11), p = 0.02 (Table 1). Conversely, the "top" set contained significantly more bulges per pre-miR than did the "depleted" set (3.53 ± 0.26 vs. 2.67 ± 0.27, p = 0.028). Similar results, with even better statistical significance, were obtained when the "top" and "next" sets were combined together, and compared against the combined "not enriched" and "depleted" sets (Table 2).

**Table 1:** Parameters scored for "top" set vs. "depleted" set of miRNAs.

	Top (n = 17)	Depleted (n = 15)	p-value
**Zone 2**			
# of stem segments	3.69 ± 0.23	3.0 ± 0.29	0.108
longest stem segment, nt.	8.41 ± 0.68	11.53 ± 1.11	0.020*
Bulges	3.53 ± 0.26	2.67 ± 0.27	0.028*

**Zone 3**			
Bulges	1.12 ± 0.21	0.8 ± 0.20	0.28

See text for details. Values are mean ± S.E.M., and p-values were calculated using two-tailed unpaired t-test with equal variance. *significant at p = 0.05 or better.

**Table 2:** Combined (top + next *vs*. not enriched +depleted) sets of miRNAs.

	Top+Next (n = 36)	Not Enriched+Depleted (n = 30)	p-value
**Zone 2**			
# of stem segments	3.53 ± 0.18	2.90 ± 0.17	0.016*
Longest stem segment, nt	8.78 ± 0.54	11.3 ± 0.67	0.0043**
Bulges	3.31 ± 0.20	2.50 ± 0.17	0.0042**
Bulges ≥ 3 nt on either side	0.20 ± 0.07	0.40 ± 0.12	0.15
Symmetrical bulges	2.17 ± 0.21	1.47 ± 0.21	0.023*
Bulges 1-0 or 1-1	2.22 ± 0.22	1.40 ± 0.22	0.010**
Bulges 1-1	1.64 ± 0.20	0.80 ± 0.19	0.0045**

**Zone 3**			
Bulges	1.08 ± 0.14	0.93 ± 0.17	0.50

**Zones 2 and 3**			
Bulges	4.39 ± 0.27	3.43 ± 0.23	0.011*
Symmetrical bulges	2.83 ± 0.25	2.03 ± 0.23	0.023*
Bulges 1-0 or 1-1	3.0 ± 0.27	1.93 ± 0.24	0.0048**
Bulges 1-1	2.14 ± 0.22	1.13 ± 0.21	0.0015**

See text for details. Values are mean ± S.E.M., and p-values were calculated using two-tailed unpaired t-test with equal variance. *significant at p = 0.05 or better. **significant at p = 0.01 or better.

The larger number of miRNAs in the combined sets permitted a more detailed statistical examination of features, including parameters that were partially correlated with each other (Table 2). The number of bulges was examined both in zones 2 and 3, separately and together, and analyzed further according to the type of bulges observed. The combined SYN-enriched set contained more bulges overall in zone 2 (but not in zone 3 alone), and contained significantly more symmetrical bulges and more small bulges containing one nucleotide on one or both sides. (Bulges having one unpaired nucleotide on each side are called 1-1 and those having only one unpaired nucleotide on one side are called 1-0) (Table 2).

Among these parameters, the one having the highest degree of statistical significance was the number of 1-1 bulges in zones 2 and 3 together (p = 0.0015). The combined SYN-enriched set exhibited 2.1 bulges of this type (1-1) per pre-miR on average, whereas those in the combined non-enriched set contained only 1.1 bulge of this type (Table 2). The overall number of bulges, of any type, also differed by an average of one bulge per pre-miR (4.4 vs. 3.4, Table 2). Thus, the increased number of bulges overall in the enriched set can be entirely explained as due to the increased number of 1-1 bulges. Across the combined SYN-enriched sets, almost all pre-miRs (34 of 36) expressed at least one 1-1 bulge, whereas of the non-enriched sets, only 20 of 30 pre-miRs expressed a 1-1 bulge [see Additional file 1]. These observations suggest that 1-1 bulges may be a particularly significant feature in a biological sense. In contrast, the number of bulges containing 3 or more bases on either side did not differ across sets (Table 2).

Several of the key tests of statistical significance in Table 2 were repeated using the non-parametric Mann-Whitney U test: The length of the longest uninterrupted stem segment gave a p-value = 0.004 by U test (cf. 0.0043 by t-test). Similarly, the number of bulges in zone 2 gave a p-value = 0.0055 (cf. 0.0042 by t-test), and the number of 1-1 bulges in zones 2 and 3 gave a p-value = 0.0014 (cf. 0.0015 by t-test). Thus, parametric and non-parametric statistical tests gave almost identical results. The trends were also detected when the structural features were correlated with the SYN enrichment ratio across all microRNAs: The SYN enrichment ratio showed a modest inverse correlation with the longest stem segment (r = -0.35), and a modest positive correlation with the number of bulges in zone 2 (r = 0.38) and the number of 1-1 bulges in zones 2 and 3 (r = 0.28). Thus, the results were not dependent on the manner of grouping the sets of microRNAs into "top", "next", "non-enriched" and "depleted".

## Discussion

Our previous study in adult mouse forebrain [10] reported that the sets of microRNAs that are enriched vs. depleted in synaptic fractions differ significantly in their tissue expression patterns, evolutionary histories, and overall expression levels. The synaptically-enriched microRNAs appear to be formed, at least in part, via local processing of pre-miR hairpin precursors occurring near synapses [10]. These previous findings imply that pre-miRs must be transported somehow to dendrites. Although one can directly test whether pre-miRs are incorporated into transport complexes, the data mining analyses reported here should provide a more detailed rationale and roadmap for experimentation [13]. Specifically, I show that the pre-miR precursors of synaptically enriched vs. depleted microRNAs differ significantly in their structural features. This provides a basis by which pre-miRs of synaptically enriched miRNAs could be preferentially transported to dendrites, via their differential association with transport complexes. Moreover, the present study identifies novel structural features of pre-miR hairpins: Precursors of SYN-enriched miRNAs contain maximal uninterrupted stem segments that are, on average, 3 nucleotides shorter than those of non-enriched miRNAs. Conversely, precursors of SYN-enriched miRNAs exhibit an extra bulge on average, consisting primarily of small symmetrical 1-1 bulges consisting of a single nucleotide on each side. These features may be important for differential transport – either via regulating the binding of proteins that mediate transport, or the binding of proteins that prevent pre-miRs from being prematurely processed into mature miRNAs during the transport process.

The structural pre-miR differences detected here should significantly affect the way that pre-miRs interact with RNA binding proteins such as dicer, TRBP, PACT, and fragile X mental retardation protein (FMRP). FMRP has been implicated in transporting mRNAs to dendrites [14-16] and can be regarded as a potential candidate to assist in pre-miR transport as well. Pre-miRs having shorter uninterrupted stem segments might be expected to bind dsRNA binding domains with less affinity, particularly if these proteins compete with each other for binding. There may be a trade-off between pre-miR structures having long stems that are optimal for immediate dicer binding and cleavage, and those having shorter stems that are optimal for transport to dendrites (a situation in which cleavage must be prevented). In agreement with this idea, the SYN enrichment ratios of microRNAs are, indeed, inversely correlated with their observed expression levels in synaptoneurosomes (r = -0.32) and forebrain homogenates (r = -0.41) (re-analysis of raw data displayed in ref. 10).

Bulges along the hairpin not only serve to interrupt stems but may play a positive role in transport by providing discrete single stranded "handles" for RNA binding proteins. The 1-1 bulge is identified here, for the first time, as a specific feature that may have biological significance, since the increased number of bulges observed in the enriched sets were accounted for almost entirely by 1-1 bulges. Nearly all (34 of 36) SYN-enriched miRNAs arise from pre-miRs that express at least one 1-1 bulge.

Selective transport of pre-miRs is likely to be only one of several factors determining the synaptic enrichment of mature miRNAs near synapses, due to the presence of additional biological events that regulate pre-miR processing [10,11,17-20]. As well, some mature miRNAs might be directly transported to dendrites by "piggybacking" in association with their mRNA targets [5,9]. However, these factors did not obscure the relationship between pre-miR structural features and synaptic enrichment (Tables 1, 2).

## Abbreviations

miRNA: microRNA; Pre-miR: microRNA hairpin precursor; SYN: synaptoneurosomes.

## Competing interests

The author declares that they have no competing interests.

## Reviewers' comments

### Reviewer's report 1

#### I. King Jordan, School of Biology, Georgia Institute of Technology

Smalheiser statistically compared the hairpin precursor structures of miRNAs (pre-miRs) that were previously found to be enriched in synaptoneurosomes to those that are non-enriched or depleted. He finds that pre-miRs enriched in synaptoneurosomes have shorter uninterrupted stem segments and more symmetrical bulges, on average, compared to the non-enriched or depleted set. These structural differences are taken as support for the hypothesis that the synaptically enriched pre-miRs are preferentially transported to dendrites.

This manuscript addresses an interesting and open question in miRNA biology. I have two main concerns regarding the data reported here and their interpretation. First of all, the structural differences between pre-miR populations analyzed here appear to be slight, and I am not yet convinced that these structural differences are statistically significant. Second, it is not clear how these structural differences lend support to the preferential transport hypothesis.

1. The author uses a parametric statistical test, the Student's t-test, to compare the structural features of pre-miRs. It is not clear whether the underlying distributions of structural features analyzed justify the use of the t-test. Furthermore, the differences revealed between sets appear to be slight and in some cases only marginally significant. It would be more conservative to use analogous non-parametric statistical comparisons, such as the Mann-Whitney U test, of these features. If both parametric and non-parametric methods yield the same results, they will be more convincing. Another more conservative approach would be to simply use simulation by randomly building sets of the same size as the top, depleted etc and measuring differences between the structural features of the simulated sets to get a distribution of differences from which to compute a test statistic. In general, since the sample sizes and differences reported here are small, there is a substantial burden of proof regarding the significance of the structural differences.

2. Different pre-miRs show different quantitative levels of enrichment. For the purposes of the statistical analysis conducted here, enrichment bins were used that grouped pre-miRs with similar enrichment or depletion levels. I couldn't help but wonder whether there was any more continuous relationship between pre-miR structural features and enrichment levels. For instance, are the number of stem segments positively correlated with enrichment or are the longest stem segment lengths negatively correlated?

3. The direct support for the preferential transport hypothesis provided by the data in this manuscript is tenuous. The seemingly best evidence for such a hypothesis, namely the correlation between relative synaptic enrichment of pre-miRs and corresponding mature miRNAs, was previously published by this same group. There does not appear to be any known, or even putative, mechanistic connection between the structural differences in enriched versus depleted pre-miRs and preferential transport. In fact, according to the author's own statements, while there is evidence that RNA-binding proteins associate with pre-miRs, there is no evidence that pre-miRs are directly associated with transport complexes. In light of the multiple levels of neuronal miRNA processing regulation, is there any reason that the structural differences uncovered here favor one mechanism (transport) over another (*e.g*. preferential processing)? Is there some reason why the structural features in the enriched set would yield pre-miRs that are preferentially transported? For instance, is there any experimental evidence which suggests that pre-miRs possessing the structural characteristics of the enriched set are preferentially bound by RNA-binding proteins such as FMRP? Is there any reason to expect that slightly shorter contiguous stems and a few more bulges would yield preferential transport?

Minor point: I was confused by the statement that "maximal uninterrupted stem segments ... correspond to sites whereby pre-miRs interact with RNA binding proteins with transport complexes." Aren't the longest uninterrupted stem segments found among the pre-miRs that are non-enriched and/or depeleted?

#### Author's response

I have now tried to deal with all of your objections.

1. I show that the t-test and Mann-Whitney U test give the same results for the most important parameters.

2. I included the correlations of SYN enrichment ratio across all mirs, with the stems and bulges, showing that they do show significant correlations in the range of 0.3–0.4.

3. You asked, Is there any reason to expect that slightly shorter contiguous stems and a few more bulges would yield preferential transport? I have tried to rewrite the paper so the answer is more obviously "yes" than before. Also, the stems are not "slightly" shorter – the average is 8-1/2 vs. 11-1/2 nucleotides in length, or about 35% difference. dsRNA binding proteins can fit 11 nucleotides in a single pocket, so 8 is likely to fit less tightly.

4. Minor point – I have now addressed more fully the "paradox" that the pre-miRs that bind less tightly to dsRNA binding proteins (e.g. to dicer) are the ones that get transported. I now acknowledge that the syn enriched mirs are also expressed at lower levels than the syn depleted mirs, which might also reflect the same pre-mir structural differences. But I do not see this as a matter of regulating expression OR regulating transport – I think that the pre-miR structural features are involved in both processes in a unified way. In the revised version, I discuss that there should be a trade off between pre-miR structures that foster rapid dicer binding and cleavage, vs. those that foster incorporation into transport complexes in which cleavage is prevented. Some proteins may actually prefer to bind shorter stems and/or may bind 1-1 bulges.

5. I also rewrote the final section where I propose specific predictions.

### Reviewer's report 2

#### Jerzy Jurka, Genetic Information Research Institute

The author uses a statistical approach to demonstrate structural differences between miRNAs enriched in a synaptic fraction of the mouse brain (SYN), relative to the miRNAs in the total forebrain homogenate. He proposes that the distinct structural features (short stems, 1-1 bulges) may be involved in transportation of precursors of the SYN-enriched microRNA to dendrites, by a number of hypothetical mechanisms.

The hypothesis may be useful for experimental researchers in the field but its presentation is somewhat confusing for a general reader. Specifically, the discussion should be shortened and rephrased to clearly differentiate between predictions based on this study and conclusions based on previously published studies. In the current version there is a disagreement between the abstract and the concluding section.

#### Author's response

I shortened the Discussion and rewrote the abstract.

## Supplementary Material

Additional file 1 **Scoring scheme for pre-miRs.** Contains the raw data used to score pre-miR features. Click here for file
